# Plasma pTau181 and pTau217 predict asymptomatic amyloid accumulation equally well as amyloid PET

**DOI:** 10.1093/braincomms/fcae162

**Published:** 2024-05-23

**Authors:** Steffi De Meyer, Jolien M Schaeverbeke, Emma S Luckett, Mariska Reinartz, Elena R Blujdea, Isabelle Cleynen, Patrick Dupont, Koen Van Laere, Jeroen Vanbrabant, Erik Stoops, Eugeen Vanmechelen, Guglielmo di Molfetta, Henrik Zetterberg, Nicholas J Ashton, Charlotte E Teunissen, Koen Poesen, Rik Vandenberghe

**Affiliations:** Laboratory for Cognitive Neurology, Department of Neurosciences, Leuven Brain Institute (LBI), KU Leuven, 3000 Leuven, Belgium; Laboratory for Molecular Neurobiomarker Research, Department of Neurosciences, Leuven Brain Institute (LBI), KU Leuven, 3000 Leuven, Belgium; Laboratory for Cognitive Neurology, Department of Neurosciences, Leuven Brain Institute (LBI), KU Leuven, 3000 Leuven, Belgium; Laboratory for Cognitive Neurology, Department of Neurosciences, Leuven Brain Institute (LBI), KU Leuven, 3000 Leuven, Belgium; Laboratory for Complex Genetics, Department of Human Genetics, KU Leuven, 3000 Leuven, Belgium; Laboratory for Cognitive Neurology, Department of Neurosciences, Leuven Brain Institute (LBI), KU Leuven, 3000 Leuven, Belgium; Neurochemistry Laboratory, Department of Clinical Chemistry, Vrije Universiteit Amsterdam, Amsterdam UMC location VUmc, 1081 HV Amsterdam, The Netherlands; Laboratory for Complex Genetics, Department of Human Genetics, KU Leuven, 3000 Leuven, Belgium; Laboratory for Cognitive Neurology, Department of Neurosciences, Leuven Brain Institute (LBI), KU Leuven, 3000 Leuven, Belgium; Nuclear Medicine and Molecular Imaging, Department of Imaging and Pathology, KU Leuven, 3000 Leuven, Belgium; Division of Nuclear Medicine, UZ Leuven, 3000 Leuven, Belgium; ADx NeuroSciences NV, 9052 Ghent, Belgium; ADx NeuroSciences NV, 9052 Ghent, Belgium; ADx NeuroSciences NV, 9052 Ghent, Belgium; Department of Psychiatry and Neurochemistry, Institute of Neuroscience and Physiology, the Sahlgrenska Academy at the University of Gothenburg, S-431 80 Mölndal, Sweden; Department of Psychiatry and Neurochemistry, Institute of Neuroscience and Physiology, the Sahlgrenska Academy at the University of Gothenburg, S-431 80 Mölndal, Sweden; Clinical Neurochemistry Laboratory, Sahlgrenska University Hospital, S-431 80 Mölndal, Sweden; Department of Neurodegenerative Disease, UCL Institute of Neurology, Queen Square, WC1N 3BG London, UK; UK Dementia Research Institute at UCL, WC1N 3BG London, UK; Hong Kong Center for Neurodegenerative Diseases, Clear Water Bay, Hong Kong, China; Wisconsin Alzheimer’s Disease Research Center, University of Wisconsin School of Medicine and Public Health, University of Wisconsin-Madison, Madison, WI 53792, USA; Department of Psychiatry and Neurochemistry, Institute of Neuroscience and Physiology, the Sahlgrenska Academy at the University of Gothenburg, S-431 80 Mölndal, Sweden; Neurochemistry Laboratory, Department of Clinical Chemistry, Vrije Universiteit Amsterdam, Amsterdam UMC location VUmc, 1081 HV Amsterdam, The Netherlands; Laboratory for Molecular Neurobiomarker Research, Department of Neurosciences, Leuven Brain Institute (LBI), KU Leuven, 3000 Leuven, Belgium; Laboratory Medicine Department, UZ Leuven, 3000 Leuven, Belgium; Laboratory for Cognitive Neurology, Department of Neurosciences, Leuven Brain Institute (LBI), KU Leuven, 3000 Leuven, Belgium; Neurology Department, UZ Leuven, 3000 Leuven, Belgium

**Keywords:** preclinical Alzheimer’s disease, blood, biomarkers, phosphorylated tau, amyloid PET

## Abstract

The dynamic phase of preclinical Alzheimer’s disease, as characterized by accumulating cortical amyloid-β, is a window of opportunity for amyloid-β–lowering therapies to have greater efficacy. Biomarkers that accurately predict amyloid-β accumulation may be of critical importance for participant inclusion in secondary prevention trials and thus enhance development of early Alzheimer’s disease therapies. We compared the abilities of baseline plasma pTau181, pTau217 and amyloid-β PET load to predict future amyloid-β accumulation in asymptomatic elderly. In this longitudinal cohort study, baseline plasma pTau181 and pTau217 were quantified using single molecule array assays in cognitively unimpaired elderly selected from the community-recruited F-PACK cohort based on the availability of baseline plasma samples and longitudinal amyloid-β PET data (median time interval = 5 years, range 2–10 years). The predictive abilities of pTau181, pTau217 and PET-based amyloid-β measures for PET-based amyloid-β accumulation were investigated using receiver operating characteristic analyses, correlations and stepwise regression analyses. We included 75 F-PACK subjects (mean age = 70 years, 48% female), of which 16 were classified as amyloid-β accumulators [median (interquartile range) Centiloid rate of change = 3.42 (1.60) Centiloids/year). Plasma pTau181 [area under the curve (95% confidence interval) = 0.72 (0.59–0.86)] distinguished amyloid-β accumulators from non-accumulators with similar accuracy as pTau217 [area under the curve (95% confidence interval) = 0.75 (0.62–0.88) and amyloid-β PET [area under the curve (95% confidence interval) = 0.72 (0.56–0.87)]. Plasma pTau181 and pTau217 strongly correlated with each other (*r* = 0.93, *P_false discovery rate_* < 0.001) and, together with amyloid-β PET, similarly correlated with amyloid-β rate of change (*r*_pTau181_ = 0.33, *r*_pTau217_ = 0.36, *r*_amyloid-β PET_ = 0.35, all *P_false discovery rate_* ≤ 0.01). Addition of plasma pTau181, plasma pTau217 or amyloid-β PET to a linear demographic model including age, sex and *APOE-ε4* carriership similarly improved the prediction of amyloid-β accumulation (ΔAkaike information criterion ≤ 4.1). In a multimodal biomarker model including all three biomarkers, each biomarker lost their individual predictive ability. These findings indicate that plasma pTau181, plasma pTau217 and amyloid-β PET convey overlapping information and therefore predict the dynamic phase of asymptomatic amyloid-β accumulation with comparable performances. In clinical trial recruitment, confirmatory PET scans following blood-based prescreening might thus not provide additional value for detecting participants in these early disease stages who are destined to accumulate cortical amyloid-β. Given the moderate performances, future studies should investigate whether integrating plasma pTau species with other factors can improve performance and thus enhance clinical and research utility.

## Teaser

De Meyer *et al*. reported that plasma pTau species predict asymptomatic amyloid-β accumulation with a moderate accuracy comparable to amyloid-β PET, suggesting that a PET scan might not provide additional value to plasma in detecting those destined to accumulate cortical amyloid.

## Graphical Abstract

**Graphical Abstract:**
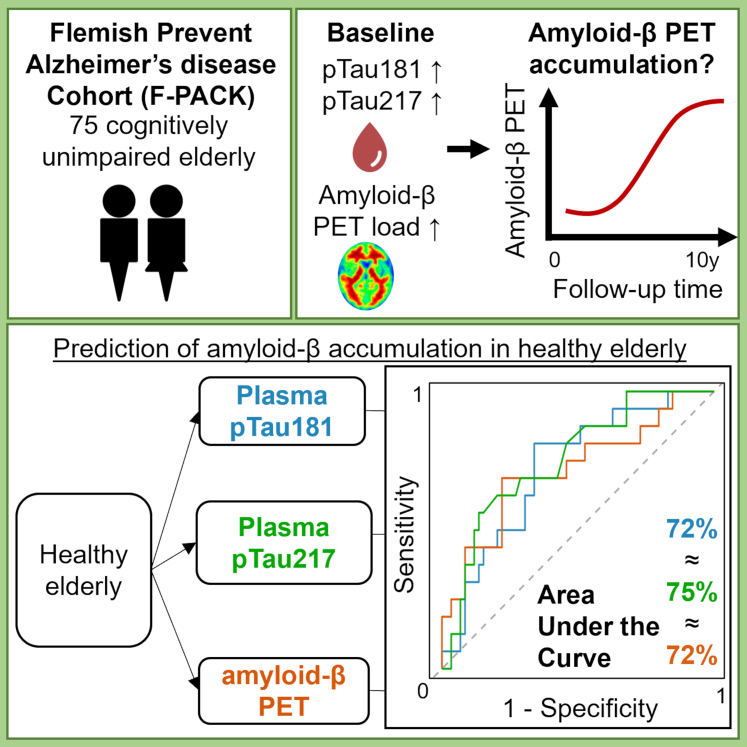

## Introduction

The amyloid cascade hypothesis states that amyloid-β (Aβ) plaques are a key driver of pathological processes in Alzheimer’s disease.^1,2^ This hypothesis has provided the impetus for clinical trials to focus on drugs promoting Aβ clearance or inhibiting Aβ production.^3^ The Alzheimer’s disease patients included in these initial trials were predominantly within prodromal or early dementia phases of the disease.^3,4^ However, Aβ positivity has been estimated to precede clinical disease onset by 20–30 years.^5^ Aβ accumulation may thus already approach a plateau within preclinical disease stages.^6–8^ Consequently, clinical trials have started to recruit cognitively unimpaired (CU) elderly who have CSF- or PET-based evidence of Aβ pathology in an attempt to maximize the therapeutic window.^3,9^ Yet, Aβ pathology has been shown to already induce downstream disease processes prior to reaching conventional Aβ positivity thresholds.^10,11^ Hence, the dynamic phase of Aβ accumulation is a unique window of opportunity for early intervention with Aβ-lowering therapies prior to the development of clinical symptoms. Biomarkers that—in addition to staging current Aβ pathology—predict future preclinical Aβ accumulation are therefore needed as they could identify clinical trial candidates in whom the efficacy of therapies in inhibiting or preventing Aβ accumulation can be assessed. To enable implementation in the large-scale screening procedures within clinical trials, such prognostic biomarkers should be cost-effective and minimally invasive. A recent study showed that plasma pTau181 is a reliable blood-based predictor of longitudinal Aβ accumulation within a median time period of 4 years when measured in predementia stages of Alzheimer’s disease.^12^ Conversely, another study in non-demented subjects with a shorter follow-up of 2 years only showed a predictive value of plasma Aβ_1-42_/Aβ_1-40_ followed by pTau217 but not for pTau181.^13^ Similarly, in a longitudinal study with a 6-year follow-up period, which examined various plasma biomarkers in asymptomatic elderly, pTau217, but not pTau181, showed good prognostic and monitoring value for brain atrophy and cognitive decline.^14^ Within that study, plasma pTau217 was also the sole biomarker demonstrating longitudinal changes.^14^ When interpreting these results, it is critical to specify the employed assay. In the cited studies, the AT270 antibody was used for plasma pTau181 quantification, which we have previously shown to cross-react with pTau175.^15^ In the current study, we used a different assay set-up based on the pTau181-specific ADx252 antibody.^15^ The ADx252-based single molecule array (Simoa) assay used here outperforms AT270-based assays in detecting Alzheimer’s disease dementia or prodromal Aβ pathology.^16–18^ Hence, the predictive value of plasma pTau181 for Aβ accumulation or other AD-related pathophysiological processes might have been underestimated compared to the highly performing pTau217 assays.^19^ Moreover, it is still unclear how the predictive values of plasma pTau181 and pTau217 for Aβ accumulation relate to that of Aβ PET and demographic or clinical information at baseline.

In this prospective longitudinal study, we directly compared the performances of plasma pTau181 quantified by an ADx252-based assay, plasma pTau217 quantified by a commercially available Simoa assay and Aβ PET ([^18^F]flutemetamol) to identify healthy elderly individuals at risk for Aβ accumulation. Furthermore, we assessed whether Aβ PET or blood-based glial fibrillary acidic protein (GFAP), neurofilament light (NfL) or Aβ_1-42_/Aβ_1-40_ as well as other Alzheimer’s disease risk factors like a polygenic risk score (PRS), education or episodic memory performance at baseline could provide additional value in the prediction of asymptomatic Aβ accumulation within a median time period of 5 years.

## Materials and methods

### Study population

Seventy-five CU older adults were selected from the F-PACK cohort based on the availability of longitudinal Aβ PET scans and baseline blood sampling. F-PACK is an academic community-recruited observational cohort of CU elderly enriched for Alzheimer’s disease risk. Recruitment followed a two-factorial genetic stratification scheme based on *APOE-ε4* and *BDNF val66met* carriership in such a way that within each 5-year age bin, the four possible combinations (carrier versus non-carrier of each gene) were equally represented. Additionally, all age bins were matched in terms of sex and education level. Other inclusion criteria included a Mini-Mental State Examination (MMSE) score ≥27, a Clinical Dementia Rating = 0 and test scores on neuropsychological assessment within published norms. Recruitment took place between March 2009 and December 2015. More details on the F-PACK cohort were described previously.^20,21^ All study participants provided written informed consent in accordance to the Declaration of Helsinki. Study protocols were approved by the Ethics Committee of University Hospitals Leuven (S51125, S65105 and S62579).

### Plasma processing and biomarker measurements

Blood was collected at University Hospitals Leuven between April 2010 and January 2018 in K2EDTA-coated polyethylene terephthalate tubes (BD Diagnostics, BD367864). All samples were centrifuged at 1200 *g* for 10 min at 4°C, and plasma was aliquoted in polypropylene cryovials (500 µL each), which were stored at −20°C for 24 h followed by long-term storage at −80°C. For each plasma sample, one aliquot was shipped to ADx NeuroSciences (Ghent, Belgium) for measurement of pTau181 and another aliquot was sent to the University of Gothenburg for pTau217 measurement. Plasma pTau181 levels were quantified in duplicate by means of a phospho-specific Simoa-based immunoassay developed by ADx NeuroSciences using Quanterix Homebrew kits and antibodies by ADx Neurosciences, i.e. incorporating ADx252 as a capture antibody, as described in detail previously.^15^ Plasma pTau217 levels were quantified in singlicate by the commercial ALZpath Simoa assay.^19^

Serum collection and measurement of GFAP, NfL, Aβ_1-42_ and Aβ_1-40_ were performed as described previously.^21^ The mean intra-assay and inter-assay coefficients of variation were equal to, respectively, 10.2% and 12.1% for pTau181, 3.2% and 6.1% for pTau217, 4.7% and 5.3% for GFAP, 3.5% and 5.3% for NfL, 3.8% and 15.5% for Aβ_1-42_ and 3.8% and 15.1% for Aβ_1-40_. All plasma and serum measurements were above the respective functional lower limits of quantification.

### Image processing and analysis

#### Structural MRI

We acquired high-resolution T_1_-weighted structural MRI scans on a 3 Tesla Philips Achieva system (Philips, Best, The Netherlands) at baseline and follow-up for all participants. All baseline scans and 40 follow-up scans were obtained using a 3D turbo field echo sequence (inversion time = 900 ms, repetition time = 9.6 ms, echo time = 4.6 ms, flip angle = 8°, field of view = 250 × 250 mm^2^, 182 slices, voxel size = 0.98 × 0.98 × 1.2 mm^3^). The remaining 35 follow-up scans were obtained using a 3D magnetization-prepared rapid gradient-echo sequence (repetition time = 6.6 ms, echo time = 3.1 ms, flip angle = 9°, field of view = 270 × 252 mm^2^, 170 slices, voxel size = 1.05 × 1.05 × 1.2 mm^3^) in the context of the multicentric AMYPAD study.^22,23^

#### Amyloid PET

Aβ PET was performed on a 16-slice Biograph PET/CT scanner (Siemens, Erlangen, Germany) at University Hospitals Leuven. Baseline Aβ PET imaging, plasma sampling and neuropsychological assessment were performed on average 6 ± 5 months apart between April 2009 and January 2019. For each participant, a follow-up Aβ PET scan was obtained between November 2018 and April 2022 with a median time interval of 5 years (range: 2–10 years) from the baseline scan. Baseline scans were obtained using either a [^18^F]flutemetamol (*n* = 57, mean activity = 149 ± 6 MBq, range: 127–162 MBq) or [^11^C]PiB (*n* = 18, mean activity = 284 ± 357 MBq, range: 175–348 MBq) tracer. All follow-up scans were performed using [^18^F]flutemetamol (*n* = 75, mean activity = 158 ± 18 MBq, range: 102–198 MBq). Tracers were injected as a bolus in the antecubital vein. PET data obtained in the 90–120 min and 40–70 min postinjection window for respectively [^18^F]flutemetamol and [^11^C]PiB were reconstructed into 6 × 5 min frames using the ordered subset expectation maximization iterative algorithm (4 iterations × 16 subsets for all baseline PET and 40 follow-up PET scans; 4 iterations × 21 subsets for 35 follow-up scans obtained within AMYPAD). Image processing was performed in Statistical Parametric Mapping 12 (SPM12, Wellcome Trust Centre for Neuroimaging, London, UK) implemented in MATLAB R2014b (Mathworks, Natick, USA). All PET images were realigned to correct for small head movements and smoothed with an isotropic Gaussian 3D kernel at 5 mm full width at half maximum. Standardized uptake value ratio (SUVR) images were constructed from the sumPET image of the first four PET frames using cerebellar grey matter as the reference region as described in detail elsewhere.^24^ Composite SUVRs were calculated in a volume of interest consisting of five bilateral cortical areas (i.e. frontal, parietal, anterior cingulate, precuneus/posterior cingulate and lateral temporal defined as AAL areas 3–10, 13–16, 23–28, 31–32, 35–36, 57–70, 81–82 and 85–90) and converted to Centiloids (CLs) for the harmonization of PET measurements across tracers.^24–26^ Aβ PET positivity implied a CL score exceeding 23.5, which corresponds to underlying Thal Phases 3 to 5.^26,27^ In order to evaluate longitudinal Aβ accumulation, Aβ rates of change were used rather than absolute changes to limit bias induced by interindividual differences in follow-up times (i.e. higher changes in longer follow-up times and vice versa). Aβ rates of change were calculated with the following formula:

$$\mathrm{Amyloid}\;\mathrm{rate}\;\mathrm{of}\;\mathrm{change}=\frac{\mathrm{Follow}\;\mathrm{up}\;\mathrm{CL}\text{-}\mathrm{Baseline}\;\mathrm{CL}}{\mathrm{Time}\;\mathrm{interval}\;(\mathrm{years})}.$$

Participants were considered to be Aβ accumulators when their Aβ rate of change was more than 1.5 interquartile ranges higher than the median CL value within the subset of CU elderly who were Aβ PET negative at both baseline and follow-up.

### PRS calculation

DNA of all subjects was genotyped using the Illumina Global Screening array covering 657 598 single nucleotide polymorphisms (SNPs).^28^ Preimputation quality control (QC) was performed using PLINK (v1.9) including a SNP call rate ≥ 0.95, minor allele frequency (MAF) ≥ 0.01 and heterozygosity (± 5 SD) with Hardy–Weinberg equilibrium threshold = 1 × 10^−6^. Imputation was performed using the Michigan Imputation Server and Haplotype Reference Consortium reference panel. Imputed data were filtered with an imputation information score >0.7 and MAF ≥ 0.01, which yielded 7 466 483 SNPs for further analysis.

A PRS for Alzheimer’s disease was calculated for all included F-PACK participants with PRSice-2 using the PRS*_noAPOE_* + *APOE_ε2+4_* approach.^23,29^ To this end, the Stage 1 summary statistics of Kunkle *et al*.^30^ served as the base file and the European individuals from the 1000 Genomes Project^31^ served as the external reference panel for clumping to remove SNPs in high linkage disequilibrium (clumping window = 250 kb, *r*^2^ = 0.1). Next, the PRS was calculated by excluding the *APOE* region (chromosome 19: 45–48.8 Mb, PRS*_noAPOE_*) and SNP inclusion threshold of 5 × 10^−8^ (genome-wide significant) followed by addition of the weighted sum of the *APOE-ε2* and *APOE-ε4* SNPs (rs429358 and rs7412) using the effect sizes from Kunkle *et al*.^30^

### Neuropsychological assessment

All F-PACK subjects underwent detailed neuropsychological assessment at baseline. We demonstrated previously that out of 10 commonly used cognitive tests, the mean Buschke Selective Reminding: Total Retention (BSRT TR) score captures the most variability in cognitive performance among CU elderly, which was therefore used as a proxy of cognitive performance in this study.^20^ The BSRT TR test is an episodic memory test in which 12 semantically and phonologically unrelated items are presented to the subject for 12 consecutive trials. Subjects are then instructed to recall all items on each trial. The average number of items recalled across the 12 trials constitutes the mean BSRT TR score.

### Statistical analysis

Statistical analyses were performed using R software (v4.2.2, The R foundation for Statistical Computing). Normality was assessed with Shapiro–Wilk tests, and group comparisons of continuous variables were performed using Mann–Whitney U-tests or *t*-tests, depending on normality. Contingency tables of binary variables were analysed using *χ*^2^ tests.

As a primary outcome analysis, we assessed the performances of baseline biomarker levels to discriminate Aβ accumulators from non-accumulators. Univariate logistic regression models with Aβ accumulator status (accumulator versus non-accumulator) as outcome variable were constructed for each biomarker separately as well as for the combination of significant predictors. Predicted probabilities of these models were entered in receiver operating characteristic (ROC) analyses using the R package ‘pROC’. Areas under the curve (AUCs) with 95% confidence intervals (CIs) were reported as measures of performance. Differences in AUCs between ROC curves were assessed using the DeLong method.^32^ For those biomarkers demonstrating significant AUCs, sensitivities and specificities as well as biomarker cut-offs were reported at maximized Youden index. In addition, the sensitivities of all three biomarkers to detect Aβ accumulators were calculated and compared when specificities were fixed at 90% and vice versa.

As a secondary outcome analysis, we evaluated the abilities of plasma pTau181, pTau217 and Aβ PET on top of demographic and clinical variables as well as other blood-based biomarkers to predict longitudinal Aβ accumulation (annualized CL rate of change) in CU elderly. To find the optimal model for the prediction of longitudinal Aβ accumulation, we first identified the variables demonstrating a significant correlation with PET-based Aβ rate of change through construction of a Pearson correlation matrix using the R package ‘corrplot’. Subsequently, we modelled longitudinal Aβ accumulation in a stepwise manner. In the first step, we established a linear demographic model including age, sex and *APOE-ε4* carriership as predictors. In a second step, we constructed single biomarker models by adding respectively baseline plasma pTau181, baseline plasma pTau217 and baseline Aβ PET to the demographic model. In subsequent steps, we constructed a multimodal biomarker model by combining pTau181, pTau217 and baseline Aβ PET followed by the addition of other variables that demonstrated significant correlations with Aβ rate of change in the correlation matrix in a stepwise manner. At each step, the additional value of the introduced variable to the model was evaluated through log-likelihood ratio tests. Akaike information criterion (AIC) and Bayesian information criterion (BIC) values were also reported as indicators of model fit along with *R*^2^ values to gauge the explained variance. AIC and BIC decreases of more than two units were considered significant.^33^ Since pTau217 has previously been suggested to change in earlier disease stages than pTau181, we also assessed the performances of all biomarkers to detect amyloid accumulators in the subset of participants who were still amyloid negative at baseline.^34^ To get a realistic *R*^2^ measure, 10-fold cross-validated training models with five repeats were computed by means of the R package ‘caret’ in order to tune the model parameter values. Reported *P*-values were corrected for multiple comparisons using the Benjamini–Hochberg false discovery rate (FDR) method, and a *P*_FDR_ < 0.05 was considered significant.

## Results

### Cohort characteristics

**Figure 1:**
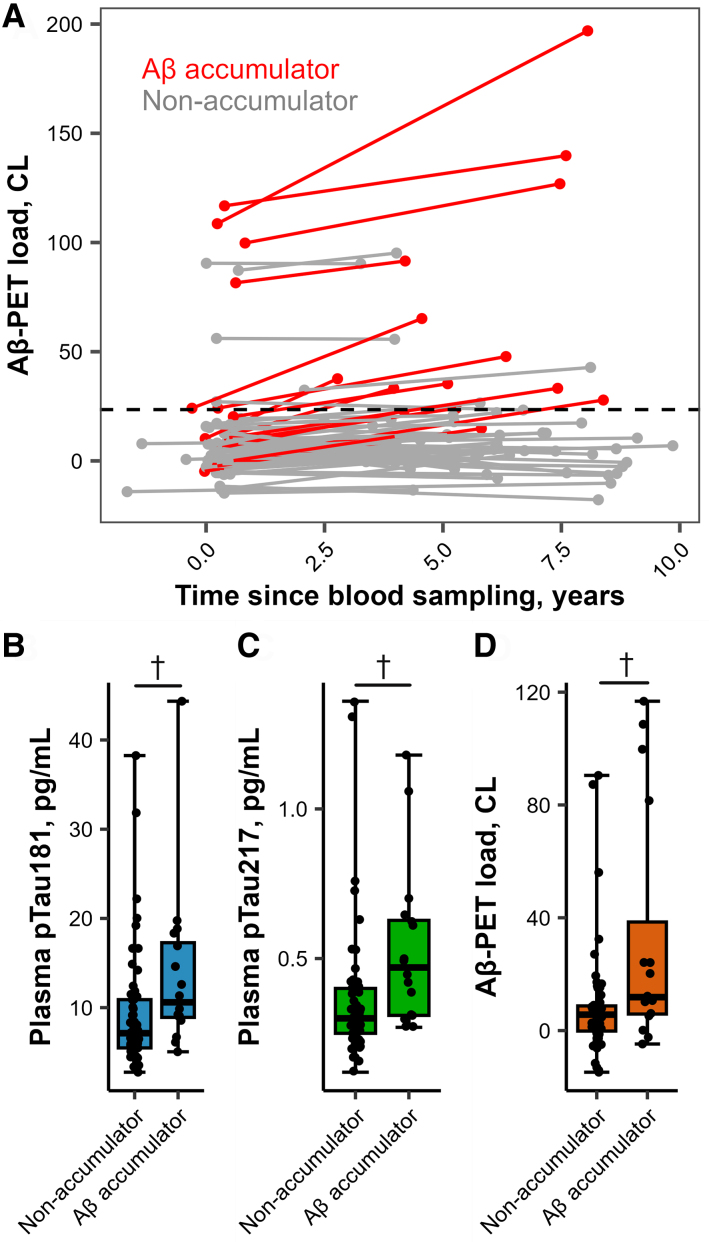
**Longitudinal Aβ accumulation and baseline biomarker levels in CU elderly.** (**A**) Spaghetti plot shows the individual longitudinal trajectories of Aβ accumulation in CU elderly (*n* = 75). (**B**, **C**) Box and whisker plots show differences in baseline plasma pTau181 levels (**B**), plasma pTau217 levels (**C**) and baseline Aβ PET load (**D**) between Aβ accumulators and non-accumulators. The middle line of the box represents the median while the lower and upper lines denote the 25th and 75th percentiles. Whiskers represent the range. Individual data points are presented on top of the plot. *P*-values were obtained using Mann–Whitney U-tests. Aβ, amyloid-β; CL, Centiloid; pTau, phosphorylated tau. ^†^*P*_FDR_ < 0.01.

Out of 75 included CU elderly, 16 were classified as Aβ accumulators based on our empirical cut-off of a 2.62 CL increase per year. Within this group of Aβ accumulators, six individuals (38%) were Aβ PET positive at baseline, which is higher than what was observed among non-accumulators (8%, *χ*^2^ = 6.31, *P* = 0.01). The remaining 10 Aβ accumulators had subthreshold Aβ levels at baseline, of which 6 became Aβ PET positive after a median follow-up period of 5 years (range 2–10 years, Fig. 1A). At baseline, Aβ accumulators did not differ from non-accumulators in age, sex, *APOE-ε4* carriership, education or episodic memory scores, and no differences in serum GFAP, NfL nor Aβ_1-42_/Aβ_1-40_ were found between the subgroups (Table 1). Median plasma pTau181 and pTau217 levels, on the other hand, were respectively 1.5- and 1.6-fold higher in Aβ accumulators than non-accumulators (Fig. 1B and C). Moreover, median baseline Aβ PET CL values were 2.1-fold higher in Aβ accumulators (Fig. 1D). Aβ accumulators also demonstrated a higher PRS than non-accumulators (*P* = 0.04).

**Table 1:** Characteristics of the study cohort

	All	Aβ accumulators	Non-accumulators	P
n (%)	75	16 (21)	59 (79)	
Baseline age, years	70 ± 6	71 ± 4	69 ± 6	0.10
Female, *n* (%)	36 (48)	8 (50)	28 (47)	1.00
APOE-ε4 carriers, n (%)	37 (49)	11 (69)	26 (44)	0.14
PRS	−0.08 ± 0.86	0.25 ± 0.63	−0.17 ± 0.90	0.04
Education, years	15 ± 3	16 ± 3	14 ± 3	0.26
Baseline Aβ PET positive, *n* (%)	11 (15)	6 (38)	5 (8)	0.01
Baseline Aβ PET, CL	6.3 [12.5]	11.9 [32.6]	5.7 [8.9]	0.008
Baseline BSRT TR,/15	8.02 ± 1.30	8.15 ± 1.51	7.99 ± 1.25	0.70
Baseline plasma pTau181, pg/mL	7.8 [5.6]	10.6 [8.4]	7.1 [5.4]	0.006
Baseline plasma pTau217, pg/mL	0.31 [0.16]	0.47 [0.32]	0.30 [0.15]	0.002
Baseline serum GFAP, pg/mL	120 [65]	136 [59]	112 [63]	0.33
Baseline serum NfL, pg/mL	15.7 [9.8]	15.2 [5.8]	15.8 [10.5]	0.94
Baseline serum Aβ_1-42_/Aβ_1-40_	0.063 ± 0.010	0.060 ± 0.009	0.063 ± 0.011	0.16
Aβ PET rate of change, CLs/year	0.61 [2.62]	3.42 [1.60]	0.28 [1.47]	<0.001
Follow-up time, years	5 [3]	5 [3]	5 [3]	0.61

Aβ accumulators were defined as subjects with an Aβ rate of change exceeding 2.62 CLs/year. Characteristics are shown for the total cohort as well as stratified by Aβ accumulator status. Continuous data are expressed as mean ± standard deviation when normally distributed and median [interquartile range] when not. Categorical data are expressed as number (%). Comparisons between cohort subgroups were made using an unpaired *t*-test (normal data), a Mann–Whitney U-test (non-normal data) or a *χ*^2^ test (categorical data).

### Performance of plasma biomarkers versus Aβ PET to discriminate Aβ accumulators from non-accumulators

**Figure 2:**
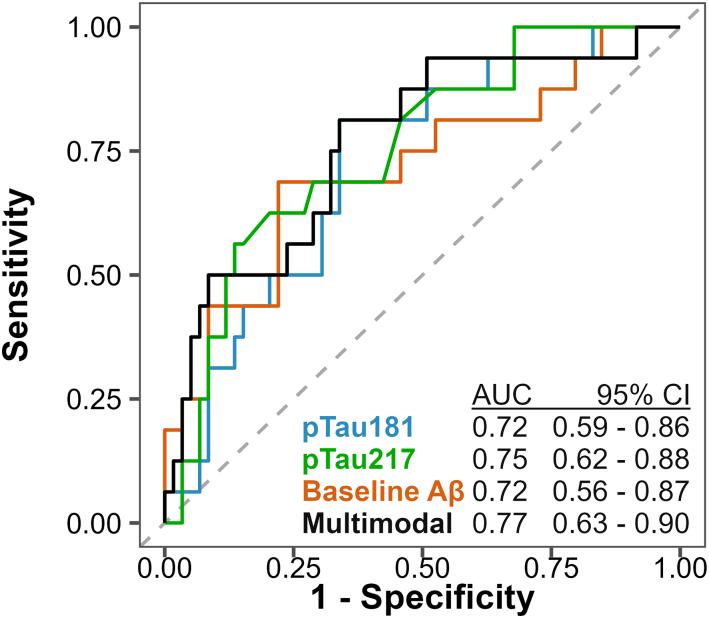
**Ability of baseline plasma pTau181, plasma pTau217 and baseline Aβ PET load to predict longitudinal Aβ accumulation in asymptomatic elderly.** ROC curves of biomarker models including plasma pTau181, plasma pTau217, Aβ PET and their combination are shown in CU elderly (*n* = 75) with Aβ accumulator status (Aβ rate of change >2.62 CLs/year, *n* = 16) as the standard of truth. AUCs with 95% CIs are superimposed at the bottom of the plot. Aβ, amyloid-β; AUC, area under the curve; CI, confidence interval; pTau, phosphorylated tau; ROC, receiver operating characteristic.

The performance of baseline plasma pTau181 to detect asymptomatic Aβ accumulators was equal to the performance of plasma pTau217 (ΔAUC = 0.03, *P*_DeLong FDR_ = 0.94, Fig. 2) as well as that of a baseline Aβ PET scan (ΔAUC = 0.00, *P*_DeLong, FDR_ = 0.94, Fig. 2). Plasma pTau217 and Aβ PET also demonstrated comparable performances in this context (ΔAUC = 0.03, *P*_DeLong FDR_ = 0.94). Combination of pTau181, pTau217 and Aβ PET in a multimodal biomarker model did not improve performance to detect Aβ accumulators compared to single biomarker models (all *P*_DeLong, FDR_ > 0.65). Other blood-based biomarkers like serum GFAP [AUC = 0.58 (95% CI 0.43–0.73)], NfL [AUC = 0.51 (95% CI 0.36–0.65)] and Aβ_1-42_/Aβ_1-40_ [AUC = 0.62 (95% CI 0.46–0.77)] could not discriminate Aβ accumulators from non-accumulators.

Within the subgroup of participants who were Aβ PET negative at baseline (*n* = 64), plasma pTau181 [AUC = 0.66 (95% CI 0.49–0.84)], pTau217 [AUC = 0.69 (95% CI 0.51–0.86)] and Aβ PET [AUC = 0.62 (95% CI 0.42–0.82)] also demonstrated comparable performances to discriminate Aβ accumulators from non-accumulators (all *P*_DeLong, FDR_ = 0.78).

At maximized Youden index, the plasma pTau181 threshold to identify Aβ accumulators was 8.6 pg/mL. This threshold corresponded to a sensitivity of 81% (95% CI 56–100%) and a specificity of 66% (95% CI 41–88%). For plasma pTau217, the threshold at maximized Youden index equalled 0.45 pg/mL, which yielded a sensitivity of 56% (95% CI 50–100%) and a specificity of 86% (95% CI 32–95%). In the model with baseline Aβ PET load as a predictor for Aβ accumulation, the threshold at Youden index was 10.2 CLs, which corresponded to a sensitivity of 69% (95% CI 38–94%) and a specificity of 78% (95% CI 58–97%).

Depending on the context of use, one may want to prioritize sensitivity or specificity. When fixing sensitivity at 90%, the specificity of baseline plasma pTau181 was 37% (95% CI 12–73%), which was comparable to the specificities of plasma pTau217 [32% (95% CI 22–67%)] and baseline Aβ PET [20% (95% CI 8–64%), all *P*_difference, FDR_ = 0.67]. When fixing the specificity at 90%, the sensitivities of plasma pTau181, pTau217 and baseline Aβ PET to detect Aβ accumulators were 31% (95% CI 0–56%), 38% (95% CI 0–75%) and 44% (95% CI 6–69%), respectively (all P_difference, FDR_ = 0.74).

### Association of clinical, demographic and biomarker variables with longitudinal Aβ accumulation

**Figure 3:**
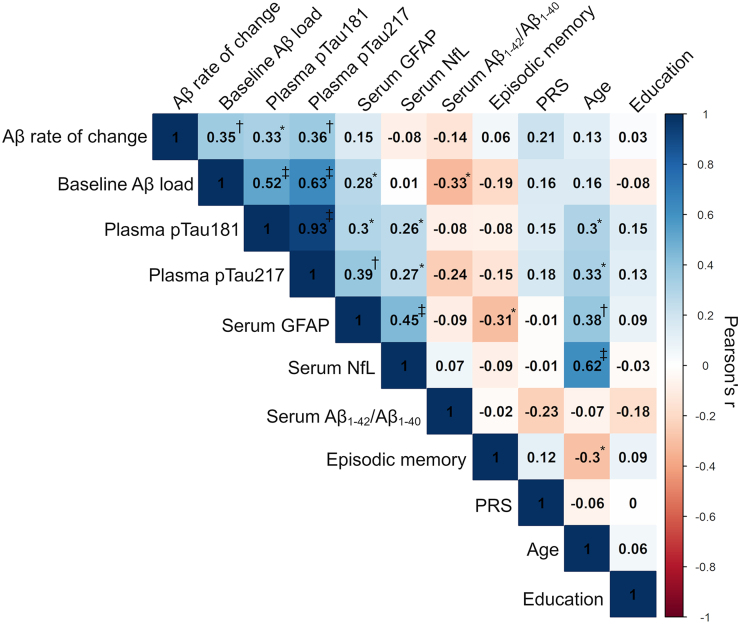
**Correlation matrix between clinical, demographic and biomarker variables.** Pearson’s correlation coefficients are superimposed on the matrix, and the colour of each box reflects the correlation strength and direction. Aβ, amyloid-β; GFAP, glial fibrillary acidic protein; NfL, neurofilament light; PRS, polygenic risk score; pTau, phosphorylated tau. *P_FDR_ < 0.05, ^†^P_FDR_ < 0.01, and ^‡^P_FDR_ < 0.001.

**Figure 4:**
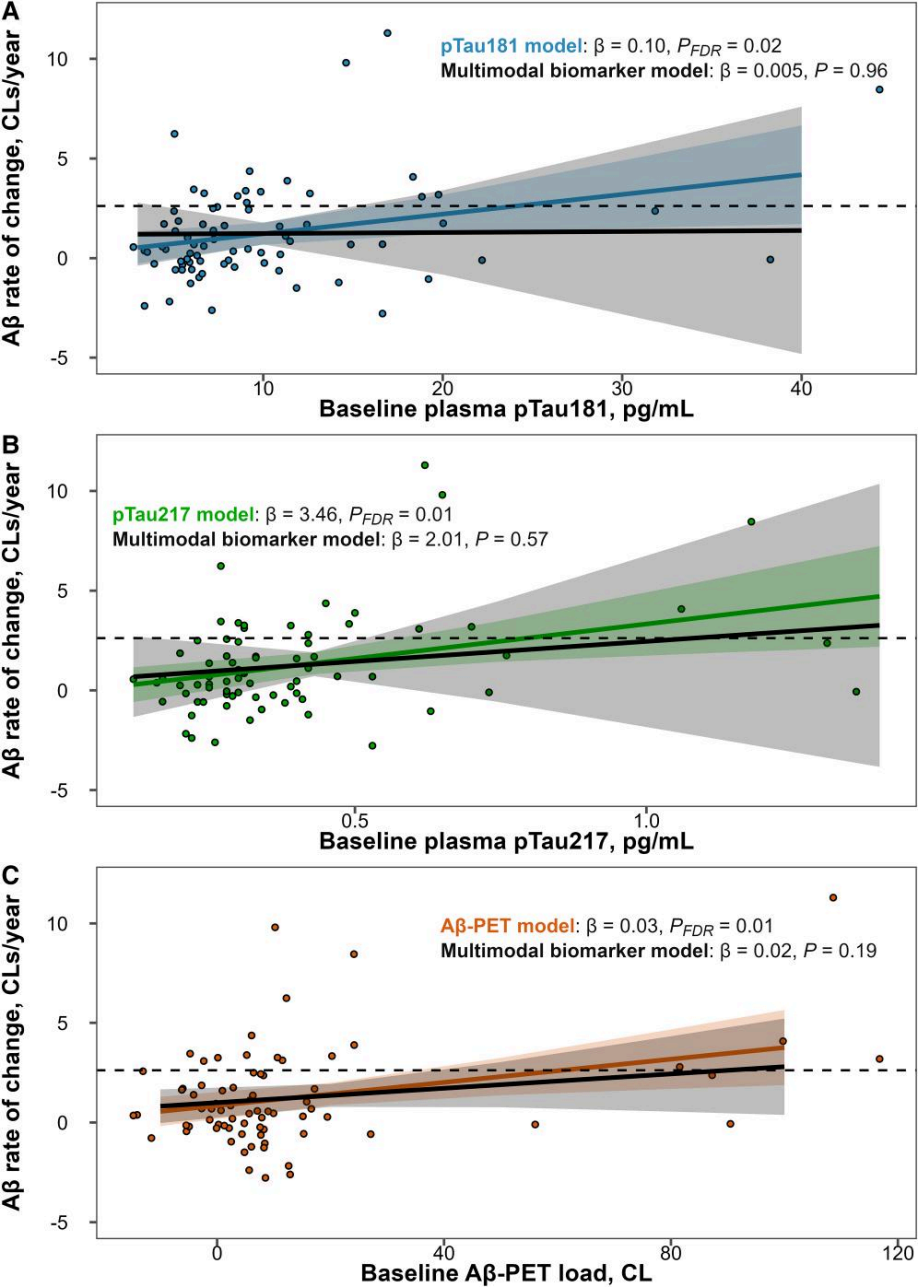
**Association of baseline biomarkers with longitudinal Aβ accumulation.** Plasma pTau181 levels (**A**), plasma pTau217 levels (**B**) and Aβ PET CLs at baseline (**C**) were plotted against the longitudinal Aβ rate of change in CU elderly (*n* = 75). Linear fits and corresponding regression coefficients are shown on top of each plot for single biomarker models of each respective biomarker as well as for multimodal biomarker models including all three biomarkers. All models were adjusted for age, sex and *APOE-ε4* carriership. Aβ, amyloid-β; CL, Centiloid; pTau, phosphorylated tau.

Plasma pTau181 (*r* = 0.33, *P*_FDR_ = 0.01) as well as plasma pTau217 (*r* = 0.36, *P*_FDR_ = 0.009) and Aβ load at baseline (*r* = 0.35, *P*_FDR_ = 0.009) were positively correlated with subsequent Aβ rates of change (Fig. 3). A weak positive correlation between the Alzheimer’s disease PRS and Aβ rate of change did not reach statistical significance (*P* = 0.07). No correlation with Aβ rate of change was observed for age, education and episodic memory performance nor for serum levels of GFAP, NfL and Aβ_1-42_/Aβ_1-40_ (all *P*_FDR_ ≥ 0.38). Of note, plasma pTau181 and pTau217 demonstrated a very strong correlation (*r* = 0.93, *P*_FDR_ < 0.001) and both moderately correlated with Aβ PET load (*r*_pTau181_ = 0.52, *P*_FDR_ < 0.001, *r*_pTau217_ = 0.63, *P*_FDR_ < 0.001). In multivariate linear regression analysis, the demographic model including age, sex and *APOE-ε4* carriership [*F*(3,71) = 1.26, *P* = 0.29] accounted for 14% of the observed variance (*R*^2^) in Aβ rate of change among CU elderly. The addition of plasma pTau181 to the demographic model improved the prediction of Aβ rate of change (*χ*^2^ = 6.1, *P*_FDR_ = 0.02) with an *R*^2^ increase of 6% (Fig. 4A and Table 2) and AIC and BIC decreases of 4.1 and 1.8 units, respectively. Addition of plasma pTau217 to the demographic model also improved the model fit (*χ*^2^ = 8.0, *P*_FDR_ = 0.01) with an *R*^2^ increase of 9% and AIC and BIC decreases of 6.0 and 3.7 units, respectively (Fig. 4B and Table 2). Addition of Aβ PET load similarly improved the model fit (*χ*^2^ = 7.9, *P*_FDR_ = 0.01) with an *R*^2^ increase of 9% and AIC and BIC decreases of 5.9 and 3.6 units, respectively (Fig. 4C and Table 2). Since plasma pTau181, plasma pTau217 and Aβ PET were the only variables demonstrating a significant association with Aβ rate of change, they were the only predictors added to the multimodal biomarker model. This multimodal biomarker model did not demonstrate a significant improvement in the prediction of Aβ rate of change relative to single biomarker models (Table 2). Moreover, in the multimodal biomarker model, plasma pTau181, pTau217 and Aβ PET load lost their individual predictive abilities (Fig. 4A–C), thus implying strong collinearity.

**Table 2:** Comparative model statistics

Model	df	AIC	BIC	*R* ^2^	Log-likelihood	*P*_demo,FDR_	*P*_multi,FDR_
Demographic model	71	354.8	366.4	0.14	−172.4		
pTau181 model	70	350.7	364.6	0.20	−169.3	0.02	0.37
pTau217 model	70	348.8	362.7	0.23	−168.4	0.01	0.37
Aβ PET model	70	348.9	362.8	0.23	−168.4	0.01	0.37
Multimodal biomarker model	68	350.8	369.3	0.19	−167.4	0.02	

Summary statistics of model performance for the five constructed multivariate linear models. *P*_demo_ and *P*_multi_ values correspond to log-likelihood ratio tests between each model and respectively the demographic and the multimodal biomarker model. AIC and BIC decreases by at least two points were considered significant.^35^

## Discussion

In this prospective longitudinal study, we showed that plasma levels of both pTau181 and pTau217 as well as Aβ PET demonstrated a similar moderate performance (AUC 0.72–0.75) to identify CU elderly in whom Aβ load will subsequently increase across a median time period of 5 years (range 2–10 years), and this is regardless of their Aβ PET status at baseline. Moreover, plasma pTau181, pTau217 and Aβ PET load correlated similarly with longitudinal Aβ rate of change, and when combined in a multimodal biomarker model, all three biomarkers lost their individual predictive abilities, implying strong collinearity.

A recent study showed that elevated plasma pTau181 levels in CU elderly are indicative for conversion to (or maintenance of) CSF- or PET-based Aβ positivity within a 10-year time frame.^35^ Since the main focus of our study was the comparison of biomarker performance to predict Aβ accumulation, the use of Aβ PET conversion status (from positive to negative) as outcome would have introduced a bias in favour of Aβ PET since subjects with a higher subthreshold Aβ PET load at baseline are more likely to cross the threshold of Aβ PET positivity, while those with a lower subthreshold Aβ PET load might not, even with steep Aβ increases. Consequently, we took a different approach and defined Aβ accumulation as a continuous outcome variable under the hypothesis that it reflects a dynamic process amenable for therapy, both in participants who are Aβ PET positive and those who are Aβ PET negative at baseline as well as in participants who may show significant Aβ accumulation but remain Aβ PET negative, even at follow-up. This is important as Aβ accumulation was previously shown to be associated with other Alzheimer’s disease hallmarks prior to reaching conventional Aβ positivity thresholds.^11,36^ To allow a direct comparison of biomarker performance, the continuous measure of Aβ rate of change was binarized in the primary outcome analysis to classify study participants into Aβ accumulators, on the one hand, and non-accumulators, on the other hand. Despite Aβ accumulators being more likely to be Aβ PET positive at baseline than non-accumulators, most individuals classifying as accumulators in the current study were Aβ PET negative at baseline (*n* = 10, 63%). Moreover, only about half of Aβ PET–negative accumulators (*n* = 6) had converted to Aβ PET positivity by the time of the follow-up visit. This further supports the notion that—in order to optimize the therapeutic window—inclusion of clinical trial participants within the dynamic phase of Aβ accumulation should not be limited to those that are already Aβ positive.

Other studies examining longitudinal Aβ accumulation in both Aβ PET–positive and Aβ PET–negative CU individuals across average time periods of respectively 4 and 6 years also found a prognostic value of plasma pTau181 for future Aβ accumulation, in line with our results.^12,37^ However, in contrast to the moderate correlation observed here (*r* = 0.33), the previous study only found a weak correlation (*r* = 0.19).^12^ Moreover, another study with a shorter follow-up time of 2 years did not show any predictive value of plasma pTau181 measurements for Aβ accumulation in CU elderly.^13^ Instead, predictive values for both plasma pTau217 and Aβ_1-42_/Aβ_1-40_ were reported. A plausible explanation of these discrepant results is the difference in used assay set-ups. In the current study, the assay set-up included the capture of tau from plasma using the ADx204 antibody in combination with detection of pTau181 using the highly T181 phospho-specific ADx252 antibody. In the previous studies, tau was captured using the Tau12 antibody—which targets the same epitope as ADx204^18^—but pTau181 was detected using the less specific AT270 antibody, which cross-reacts with pTau175. As mentioned above, the ADx252-based Simoa assay outperforms AT270-based assays in distinguishing Alzheimer’s disease patients from healthy controls as well as in detecting Aβ pathology in clinical stages.^16,17^ Moreover, in these studies, ADx252-based—but not AT270-based—pTau181 measurements performed equally to immunoassay measurements of plasma pTau217. We now showed that ADx252-based pTau181 measurements also perform similarly to pTau217 measurements with respect to the prediction of Aβ accumulation. Although plasma pTau217 did exhibit slightly higher numerical performance than pTau181 in terms of identifying Aβ accumulators as well as predicting longitudinal Aβ accumulation, this difference did not reach statistical significance.

The comparable performances of plasma pTau181, pTau217 and Aβ PET to predict future Aβ accumulation suggest that plasma pTau species might provide a more accessible alternative for large-scale screenings to enrich clinical trials with participants that are at risk for Aβ accumulation. This is especially true for early prevention trials with Aβ-lowering drugs, which are likely to be most efficacious in individuals in early Aβ phases. Consequently, clinical trials that recruit asymptomatic individuals with underlying Alzheimer’s disease pathology have been initiated. Whereas clinical trials including symptomatic patients have traditionally focussed on clinical progression as a primary outcome, this paradigm shift towards earlier disease stages warrants new monitoring strategies targeting earlier (biological) processes. Since Aβ accumulation is a biological process that constitutes the earliest pathological change in Alzheimer’s disease that can be reliably detected using biomarkers, evaluating therapy effect based on Aβ rate of change might be a more suited approach in these asymptomatic individuals. So far, three studies have included Aβ accumulation as the primary (AHEAD A3) or secondary (A4, SKYLINE) outcome measure. In this context, we found that combining plasma pTau species with Aβ PET did not increase performance for detecting Aβ accumulators relative to each biomarker separately. Together with the observed strong correlations between pTau181 and pTau217 levels in plasma and the comparable correlations of both pTau species with Aβ PET load as well as the lack of predictive value of either of these biomarkers when combined in a multimodal biomarker prediction model, this suggests that Aβ PET load, pTau181 and pTau217 reflect similar pathophysiological processes. Of note, the observed performances of either single biomarker model (pTau181, pTau217 and Aβ PET) were only modest, and when evaluating sensitivities at fixed specificities of 90% or vice versa, they all dropped below 44%. Hence, there is a need to identify factors that could further improve the accuracy of the current plasma pTau assays before we can reliably predict Aβ accumulation in a clinical trial setting within individuals in this early Alzheimer’s disease stage. In the current study, serum biomarkers such as GFAP, NfL and Aβ_1-42_/Aβ_1-40_ could not discriminate Aβ accumulators from non-accumulators and showed no significant associations with longitudinal Aβ accumulation and were thus unlikely to sufficiently increase the predictive value of plasma pTau species. Future studies should explore whether factors reflecting other early pathophysiological processes (e.g. synaptic markers or pTau231^14,34^) can further improve this performance.

The main limitation of the current study is the relatively small sample size of 75 participants. This might have prevented us from obtaining significance for some of the weaker associations (i.e. Alzheimer’s disease PRSs were higher in accumulators than non-accumulators, but showed no continuous association with Aβ PET CL rate of change, unlike findings from our previous report in a larger F-PACK subset^23^). Moreover, a larger sample size would have allowed subgroup analyses, which could have shed light on the distinct timeframes in which plasma pTau181 and pTau217 versus Aβ PET are predictive for Aβ accumulation. Secondly, Aβ PET was performed using two different PET tracers, among and within subjects, which might have introduced bias despite our efforts to harmonize measurements through CL conversion. Moreover, time intervals between baseline and follow-up PET scans were not constant among individuals, but varied from 2 to 10 years. We corrected for this variability by using rates of change rather than absolute changes, but Aβ rates of change are not constant across the complete trajectory of Aβ accumulation.^38^ Therefore, differences in temporal offset between the initial Aβ PET scan and the onset of the dynamic Aβ phase is a possible source of bias as longer follow-up times increase the possibility of the onset of Aβ accumulation occurring during follow-up. However, since follow-up times were not higher among Aβ accumulators than non-accumulators, this bias might be limited in our study. Future studies in larger cohorts with consistent follow-up periods should evaluate whether a baseline Aβ PET scan (or other biomarkers) might provide added value when implemented in a two-step screening procedure where CU elderly with elevated plasma pTau levels are submitted to further testing. To enable such studies, standardized threshold values are needed in order to ensure consistent and reliable interpretation of the results.

## Conclusion

We showed that baseline plasma pTau181 and pTau217 levels predict Aβ accumulation in the asymptomatic phase of Alzheimer’s disease equally well as baseline Aβ PET. Moreover, in a multimodal biomarker model, none of the three biomarkers remained a significant predictor of future Aβ accumulation, thus suggesting they might convey overlapping information such that their combined use might not yield additional value in predicting early Aβ deposition. However, the observed performances for plasma pTau181 as well as pTau217 were only moderate, so future studies should investigate whether combining plasma pTau181 or pTau217 with other factors—whether in panels or in a two-step manner—would allow for further improvement in performance and thus enhanced clinical and research utility.

## Data Availability

Data are available from the corresponding author on reasonable request for the sole purpose of recreating the procedures or results presented in the current study. Such data transfer will be regulated through a material transfer agreement.
